# Determinants of Burnout in Acute and Critical Care Military Nursing Personnel: A Cross-Sectional Study from Peru

**DOI:** 10.1371/journal.pone.0054408

**Published:** 2013-01-14

**Authors:** Elizabeth Ayala, Andrés M. Carnero

**Affiliations:** 1 Critical Care Department, Hospital Central de la Fuerza Aérea del Perú, Lima, Peru; 2 Postgraduate School, Universidad Peruana Cayetano Heredia, Lima, Peru; The University of Hong Kong, Hong Kong

## Abstract

**Background:**

Evidence on the prevalence and determinants of burnout among military acute and critical care nursing personnel from developing countries is minimal, precluding the development of effective preventive measures for this high-risk occupational group. In this context, we aimed to examine the association between the dimensions of burnout and selected socio-demographic and occupational factors in military acute/critical care nursing personnel from Lima, Peru.

**Methods and Findings:**

We conducted a cross-sectional study in 93 nurses/nurse assistants from the acute and critical care departments of a large, national reference, military hospital in Lima, Peru, using a socio-demographic/occupational questionnaire and a validated Spanish translation of the Maslach Burnout Inventory. Total scores for each of the burnout dimensions were calculated for each participant. Higher emotional exhaustion and depersonalisation scores, and lower personal achievement scores, implied a higher degree of burnout. We used linear regression to evaluate the association between each of the burnout dimensions and selected socio-demographic and occupational characteristics, after adjusting for potential confounders. The associations of the burnout dimensions were heterogeneous for the different socio-demographic and occupational factors. Higher emotional exhaustion scores were independently associated with having children (p<0.05) and inversely associated with the time working in the current department (p<0.05). Higher depersonalization scores were independently associated with being single compared with being divorced, separated or widowed (p<0.01), working in the emergency room/intensive care unit compared with the recovery room (p<0.01), and inversely associated with age (p<0.05). Finally, higher personal achievement scores were independently associated with having children (p<0.05).

**Conclusion:**

Among Peruvian military acute and critical care nursing personnel, potential screening and preventive interventions should focus on younger/less experienced nurses/nurse assistants, who are single, have children, or work in the most acute critical care areas (e.g. the emergency room/intensive care unit).

## Introduction

Burnout is a persistent condition resulting from chronic exposure to occupational stress at levels beyond the individual's coping capacity [1]. It is highly prevalent in the health professions, particularly among nurses [2], who may be rendered more vulnerable by the demanding nature of their profession [3], [4], [5] and the prevailing adverse professional scenario [6], [7]. Burnout is an increasingly important public health problem, which has a significant impact on the nurses' health and well-being, and the quality and costs of healthcare. It leads to emotional exhaustion, depersonalisation and a perception of inefficacy at work [1], [8]. Moreover, it is also associated with a wealth of psychosomatic symptoms [8], [9], impaired quality of life [10], and may predispose to mental [11], [12] and physical disease [12]. In addition, burnout negatively affects the nurses' work performance [13], [14], patient safety [14], and patients'/relatives' satisfaction [15], [16]; increases the risk of occupational injuries [17], and predisposes to absenteeism [18], job dissatisfaction [19], and nurse turnover [20]. Finally, burnout may predispose other nurses to experience the condition [21], establishing a self-perpetuating vicious cycle leading to poor quality of care and increased organizational costs.

The prevalence and impact of burnout is not uniform across the different nursing specialties [22], [23] and/or work settings [24], [25]. In terms of nursing specialties, acute and critical care nurses seem to be at a substantially high risk of burnout [23], [26], [27], due to the exposure to a highly stressful environment in the emergency [28], [29] and intensive care unit [30], [31], [32], [33]. Furthermore, the impact of burnout on acute and critical care nurses may be exacerbated by the current shortage of critical care professionals [34]. In terms of work setting, military nurses may constitute a group at high-risk of burnout [35], [36], [37]. This may be ascribed to the demanding job of military nursing, the altruist military philosophy, the stigma associated with mental health services, and the differing characteristics between military and civilian nurses [24], [37], [38], [39]. In addition, burnout may have a greater impact in the military setting, since military institutions have little flexibility for improving nurse retention [35], [40]. Thus, military acute and critical care nurses constitute a group especially susceptible to burnout and its detrimental effects. Nevertheless, few studies have evaluated the correlates of burnout in military nurses (and even fewer in military critical care nurses), and have been conducted almost exclusively in affluent countries [24], [35], [36], [37], [41], [42]. This is not a trivial issue, since the cultural, socioeconomic and political context of developing countries may result in an increased prevalence and toll of burnout, and limit the applicability of findings from other contexts.

In Peru, there is a dearth of information on burnout among military acute and critical care nursing personnel. Nevertheless, several factors may result in an increased prevalence and impact of burnout in this occupational group: the poor economic context may result in stressful working conditions [7], with poor resources and high demands [43]; in addition the continued military interventions in the war against terrorism and the control of social conflicts may result in an increased work burden. Thus, studies on military acute and critical care nurse burnout and its determinants in Peru are needed in order to improve our understanding of the condition, and develop potential preventive interventions for this high-risk occupational group. In this context, we aimed to identify the socio-demographic and occupational characteristics associated with the dimensions of burnout in acute and critical care nursing personnel from a large military hospital in Lima, Peru.

## Methods

### Study Design and Setting

We conducted a cross-sectional study in the nursing personnel of the acute and critical care departments of Hospital Central de la Fuerza Aérea del Perú (HFAP), a large, national reference, tertiary (highest complexity) military hospital in Lima, Peru. HFAP serves active and retired military personnel and their close relatives (spouse and children), offering care in 24 medical specialties, including acute and critical care. Its critical care department is comprised by several sub-sections, including: the emergency department, the operating room and recovery room, the intermediate care unit and the critical care unit. Each department has a nursing team composed of a chief nurse, clinical nurses and nurse assistants, who work in 6–12 hour rotating shifts to provide continuous acute and critical care to patients. In Peru, nursing personnel is comprised by nurses and nurse assistants. Nurses must complete a 5-year BSc university programme and approve a Licensure Thesis in order to obtain license to practice. Nurse assistants typically complete a 3-year training programme in a superior institute; and perform duties to aid indirectly in patient care, under the supervision of nurses.

### Study Participants

The study included all the nursing personnel who worked in the hospital's acute and critical care departments (intensive care unit, intermediate care unit, operating room, recovery room, and emergency department), had at least 1 year of experience in acute and critical care nursing practice, and were willing to participate in the study. We did not include nursing personnel with less than 1 year of clinical nursing work experience in order to ensure that they had experienced a minimum exposure to the demands of critical care. Potentially eligible participants were identified from the hospital's administration records. Of a total 120 nurses and nurse assistants working in the hospital's critical care departments, 110 (91.7%) fulfilled the study selection criteria; and, of these, 93 (84.5%) agreed to participate in the study.

### Data Collection and Variable Definitions

The study consisted of an anonymous, structured, self-administered, paper-based survey, conducted between May-June 2011, which was comprised by two parts: an initial socio-demographic/occupational questionnaire and the Maslach Burnout Inventory.

The socio-demographic/occupational questionnaire included 10 questions related to socio-demographic data (gender, age, marital status, children) and occupational data [occupation (nurse or nurse assistant), work department, contractual status (tenured or contracted), number of years working in the current department and in the institution, and the presence of an additional job].

The Maslach Burnout Inventory (MBI) is the gold standard for detecting burnout [1], [8]. It has been used in a wide range of professions and settings, and has been translated to many languages. Furthermore, it has been shown to perform adequately and have a stable factorial structure among nurses from different populations and contexts [44], including critical care [45]. The MBI Human Services Survey (MBI-HSS) is a 22-item questionnaire designed to assess the presence of burnout in health care providers, which contains three subscales, each evaluating one of the constructs of burnout: emotional exhaustion (9 items), depersonalisation (5 items) and personal achievement (8 items). Each subscale is comprised by several items, each of which describes a particular feeling/attitude towards work, and is rated by the respondent on a 7-point Likert scale according to the frequency with which he/she has experienced such emotion/attitude (ranging from 0 =  “Never” to 6 =  “Every day”). The EE subscale evaluates the feeling of having depleted one's emotional resources to deal with the situations arising at work. The DP subscale quantifies the attitude of distancing from other persons. Finally, the PA subscale reflects the extent to which one recognises the value of its own work. Ratings for each item are summed to calculate the score for each subscale. As suggested by its authors, burnout is a multidimensional phenomenon, and the scores for the three dimensions should not be combined in a total score. For both the EE and DP subscales, higher scores imply a higher level of burnout; while for the PA subscale, a lower score indicates a higher level of burnout. We used a validated Spanish version of the MBI-HSS (permission obtained from the publisher, Mind Garden, Inc. and CPP, Inc.), which has been reported to have a satisfactory Cronbach's alpha for personal achievement (PA, α = 0.71) and emotional exhaustion (EE, α = 0.85), and moderate for depersonalization (DP, α = 0.58) [46].

Although, as mentioned above, the MBI-HSS has been shown to perform adequately across different populations, considerable score variability has been reported between populations, leading to the recommendation to avoid using cut-off points derived from foreign populations [47]. Thus, because reference scores have not been derived for Peruvian health professionals, we analysed EE, DP and PA scores as continuous variables. This obviated the need of using reference cut-off points derived in other countries/occupational groups, but also precluded defining “high” scores for each of the burnout dimensions, and thus, estimating the prevalence of burnout. Finally, although some evidence suggests that personal achievement is not a separate burnout dimension [43], [48], because this view is still controversial, we evaluated the socio-demographic and occupational factors associated with all three dimensions of burnout.

### Statistical Analysis

Statistical analyses were performed using Stata/IC® 11.0 (StataCorp LP, College Station, Texas, 2011, US). For the descriptive analysis, we calculated the central tendency and dispersion statistics for each variable and evaluated their distribution using graphical and numerical methods, as well as formal statistical tests. For the bivariate analysis, we compared the mean scores for each subscale by socio-demographic and occupational characteristics using Student's t test for dichotomous categorical variables and simple linear regression with robust variance estimation (see below) for polytomous categorical variables and numerical variables. Finally, for the multivariable analysis, we used multiple linear regression with robust variance estimation (see below) to estimate the mean difference in the MBI subscale scores [and 95% confidence intervals (95% CI)] by socio-demographic and occupational variables, after adjusting for potential confounders. We tested the model assumptions using both graphical residual analysis and formal statistical tests. Although the MBI subscale scores were not normally distributed, it has been shown that Student's t tests and multiple linear regression are robust to moderate/severe departures from normality in the setting of moderate (i.e. not very small) sample sizes [49], which was the case for this study. A more critical assumption is that of homoscedasticity, particularly for inferential purposes. Because we found evidence of heteroskedasticity, we used a heteroskedasticity consistent standard error (HCSE) estimator for linear regression [50]. Specifically, we used the “HC3” HCSE estimator, which has been shown to be the most consistent and unbiased estimator, particularly for small sample sizes without influential observations [50], [51]. All statistical tests were two-sided, and a significance level of 0.05 was considered relevant.

### Ethical Considerations

The study received ethical approval from the Institutional Review Board (IRB) of Hospital Central de la Fuerza Aérea del Perú in Lima, Peru. Written informed consent was obtained from all of the study participants.

## Results

A total of 93 nurses/nurse assistants completed the survey (84.5% response proportion). They were predominantly female (94.6%), had a mean age (standard deviation) of 42.7 (7.9) years, were mostly married or cohabitating (60.2%) and had children (69.9%). Regarding work, most were nurses (58.1%), were tenured (92.5%), and had worked more than 5 years in their current department (65.6%). Finally, 30.1% of the nursing personnel had an additional job outside HFAP. Table 1 shows the socio-demographic and occupational characteristics of the study population.

**Table 1:** Socio-demographic and work-related characteristics of the study population.

Characteristic	N (%)
Gender	
Female	88 (94.6)
Male	5 (5.4)
Age (years)†	42.7 (7.9)
Marital Status	
Married/Cohabitating	56 (60.2)
Single	31 (33.3)
Other	6 (6.5)
Children	
No	28 (30.1)
Yes	65 (69.9)
Occupation	
Nurse	54 (58.1)
Nurse assistant	39 (41.9)
Department	
Emergency/ICU	45 (48.4)
OR	23 (24.7)
Recovery	13 (14.0)
Intermediate care	12 (12.9)
Contract Status	
Tenured	86 (92.5)
Contract	7 (7.5)
Years in the institution†	16.1 (7.5)
Years in the current department‡	9.0 (8.0)
Additional work	
Yes	28 (30.1)
No	65 (69.9)

† Mean (standard deviation), ‡ Median (Interquartile Range).

OR: Operating Room, ICU: Intensive Care Unit.

Table 2 presents selected summary statistics for each of the MBI subscales scores. The median score and interquartile range (IQR) for the EE, DP and PA subscales was 9 (IQR: 11), 2 (IQR: 6), and 42 (IQR: 8), respectively. EE and DP scores exhibited a marked positive skew, with a larger variation in the scores on the upper-end of the scale; while PA scores exhibited a moderate negative skew, with a greater spread in the scores on the lower-end of the scale. Finally, the EE and PA subscales showed an adequate Cronbach's alpha (0.85 and 0.70, respectively), while the DP subscale exhibited a moderate Cronbach's alpha (0.56).

**Table 2:** Distribution of Maslach Burnout Inventory subscale scores.

Subscale	N	Mean	SD	Median	IQR	Percentiles		Cronbach's
						33.3th	66.7th	alpha
Emotional Exhaustion	93	11.9	9.0	9.0	11.0	6.0	16.0	0.85
Depersonalisation	93	3.9	4.4	2.0	6.0	1.0	6.0	0.56
Personal Achievement	93	41.0	6.4	42.0	8.0	39.0	45.0	0.72

SD: Standard deviation, IQR: Interquartile range.

Table 3 shows the mean MBI subscales scores (and standard deviations) by socio-demographic and occupational characteristics. Having children was associated with a higher mean EE score (p<0.05), while the number of years working in the current department was associated with a lower mean EE score (p<0.01). Regarding the DP scores, male gender was associated with a higher mean DP score (p<0.05), while an older age was associated with a lower mean DP score (p<0.05). On the other hand, being divorced, separated or widowed was associated with a lower mean DP score (p<0.01) compared with being single, Also, working in the recovery room, compared with the emergency room/intensive care unit (p<0.01) was associated with a lower DP score. Finally, PA scores were not significantly associated with any of the socio-demographic or occupational factors.

**Table 3:** Mean Maslach Burnout Inventory subscale scores (and standard deviation) by socio-demographic and work-related characteristics.

Characteristic	Emotional Exhaustion		Depersonalisation		Personal Achievement	
	Mean (SD)	*P* value	Mean (SD)	*P* value	Mean (SD)	*P* value
Gender		0.26		0.04		0.53
Male	18.8 (12.6)		8.4 (4.5)		40.9 (6.4)	
Female	11.5 (8.7)		3.7 (4.3)		43.0 (6.6)	
Age (years)†	−0.16	0.10	−0.12	0.03	0.07	0.38
Marital Status		0.36		<0.01		0.31
Married/Cohabitating	12.9 (9.7)		3.7 (4.0)		40.6 (6.4)	
Single	10.5 (7.8)		4.8 (5.2)		41.3 (6.7)	
Other	9.5 (7.0)		1.0 (1.3)		43.8 (4.4)	
Children		0.04		0.80		0.10
No	9.2 (7.0)		4.1 (5.0)		42.4 (4.4)	
Yes	13.0 (9.5)		3.8 (4.1)		40.4 (7.0)	
Occupation		0.21		0.24		0.85
Nurse	10.8 (8.1)		3.4 (4.3)		41.1 (5.7)	
Nurse assistant	13.3 (10.0)		4.6 (4.6)		40.8 (7.2)	
Department		0.21		<0.01		0.25
Emergency/ICU	11.4 (9.3)		4.4 (4.7)		40.7 (5.2)	
OR	10.6 (8.0)		4.2 (4.6)		40.0 (7.3)	
Recovery	10.8 (7.7)		0.8 (1.3)		44.2 (5.7)	
Intermediate care	17.4 (9.5)		5.0 (3.8)		40.6 (5.4)	
Contract Status		0.47		0.18		0.48
Tenured	11.7 (9.1)		3.7 (4.3)		41.2 (6.1)	
Contract	14.1 (7.9)		6.6 (5.1)		38.4 (9.5)	
Years in current department†	−0.37	<0.01	0.00	0.98	0.09	0.35
Additional work		0.91		0.32		0.11
Yes	11.7 (8.0)		4.7 (5.1)		42.5 (5.4)	
No	11.9 (9.4)		3.6 (4.1)		40.4 (6.7)	

† Simple linear regression coefficient associated with a 1 year increase.

SD: Standard deviation, OR: Operating Room, ICU: Intensive Care Unit.

Table 4 shows the multiple linear regression coefficient for each of the burnout dimensions (β, i.e. the difference in the mean scores for each dimension) and 95% confidence intervals by socio-demographic and work-related characteristics, adjusted for gender, age and occupation. Regarding the burnout dimensions, each of the MBI subscales was associated with different socio-demographic and occupational factors. EE scores were independently associated with having children (p<0.05) and inversely associated with the time working in the current department (p<0.05). On the other hand, DP scores were independently and inversely associated with age (p<0.05), being divorced, separated or widowed compared with being single (p<0.01), and working in the recovery room compared with the emergency room/intensive care unit (p<0.01). Finally, PA scores were independently associated with having children (p<0.05).

**Table 4:** Adjusted multiple linear regression coefficient (β) and 95% confidence intervals (95% CI) for the scores of each of the Maslach Burnout Inventory subscales by socio-demographic and work-related characteristics†.

Characteristic	Emotional Exhaustion		Depersonalisation		Personal Achievement	
	β (95% CI)	*P* value	β (95% CI)	*P* value	β (95% CI)	*P* value
Gender		0.31		0.06		0.43
Male	6.7 (−6.2, 19.6)		4.3 (−0.2, 8.8)		2.5 (−3.7, 8.6)	
Female	0.0 (Reference)		0.0 (Reference)		0.0 (Reference)	
Age (years)‡	−0.1 (−0.4, 0.1)	0.21	−0.1 (−0.2, −0.0)*	0.04	0.1 (−0.1, 0.2)	0.34
Marital Status		0.31		<0.01		0.33
Married/Cohabitating	2.8 (−0.9, 6.4)		−1.0 (−3.1, 1.2)		−1.1 (−4.1, 1.9)	
Single	0.0 (Reference)		0.0 (Reference)		0.0 (Reference)	
Other	0.5 (−6.5, 7.5)		−3.1 (−5.1, −1.1)		2.1 (−2.7, 6.8)	
Children		0.02		0.77		0.04
No	0.0 (Reference)		0.0 (Reference)		0.0 (Reference)	
Yes	4.0 (0.5, 7.5)*		−0.3 (−2.4, 1.8)		−2.6 (−5.1, −0.1)*	
Occupation		0.20		0.20		0.84
Nurse	0.0 (Reference)		0.0 (Reference)		0.0 (Reference)	
Nurse assistant	2.6 (−1.4, 6.5)		1.2 (−0.6, 3.0)		−0.3 (−3.1, 2.5)	
Department		0.29		<0.01		0.35
Emergency/ICU	0.0 (Reference)		0.0 (Reference)		0.0 (Reference)	
OR	−1.0 (−5.7, 3.6)		−0.2 (−2.7, 2.3)		−0.5 (−3.8, 2.8)	
Recovery	0.8 (-4.6, 6.2)		−2.9 (−4.5, −1.2)**		3.5 (-0.7, 7.7)	
Intermediate care	5.6 (−0.9, 12.1)		0.4 (−2.0, 2.8)		−0.1 (−4.2. 4.0)	
Contract Status		0.85		0.68		0.50
Tenured	0.0 (Reference)		0.0 (Reference)		0.0 (Reference)	
Contract	−0.7 (−8.4, 6.9)		1.0 (−3.6, 5.7)		−3.2 (−12.4, 6.1)	
Years in current department‡	−0.3 (−0.6, −0.0)*	0.04	0.1 (−0.1, 0.2)	0.27	0.1 (−0.1, 0.3)	0.52
Additional work		0.71		0.47		0.14
Yes	0.7 (−3.3, 4.8)		0.8 (−1.4, 3.0)		2.0 (−0.7, 4.7)	
No	0.0 (Reference)		0.0 (Reference)		0.0 (Reference)	

† Adjusted for gender, age and occupation (nurse/nurse assistant).

‡ Multiple linear regression coefficient associated with a 1 year increase.

OR: Operating Room, ICU: Intensive Care Unit.

* p<0.05.

** p<0.01.

## Discussion

This study shows that several socio-demographic and occupational factors are independently associated with the dimensions of burnout among Peruvian military critical care nurses. Also, each dimension is associated with different socio-demographic and occupational characteristics.

We found no association between gender and the burnout dimensions, although such analysis is limited by the scarce number of male nurses/nurse assistants (n = 5). Previous studies among military nurses have reported conflicting results [35], [41], [52], which may in part be explained by the fact that these associations may be confounded by several factors (such as occupational role and hierarchical position, among others) [1], [8].

Age was independently and inversely associated with DP scores, in agreement with previous reports [41], [52]. A closely related variable, the time working in the current department, was independently and inversely associated with EE scores, as reported by most [41], [52], but not all [35], [42] previous studies. These associations may be explained by the development of better coping strategies by more experienced nurses [53], as both variables are closely related with professional experience. Alternatively, this association may reflect a “survival bias” (as this study was cross-sectional) [1], [8]. Longitudinal studies are needed to elucidate this association in military acute and critical care nurses.

Having children was associated with greater EE and lower PA scores, as a previous study reported [52]. Studies conducted in civilian nurses have reported that having children is positively associated with burnout, probably by generating additional responsibilities for the nurse and generating family-work conflicts [53], [54]. This may be particularly true for female nurses in Peru, where traditional cultural beliefs assign the child-rearing responsibility exclusively to women.

Previous studies have reported that being married/having a stable partner is not associated with burnout or its dimensions [41], [42], and that it is associated with lower EE scores [52]. In this study, we found that being married and cohabitating were not associated with a greater score in any of the burnout dimensions, but being divorced, separated or widowed was associated with lower DP scores, compared with single nurses (unfortunately, small counts did not allow for further exploration), in agreement with previous data [1]. Although it has been reported that being married or cohabitating may result in greater responsibilities/time demands, inducing family-work conflicts and leading to burnout [53], [54], an alternative scenario has also been proposed, in which a supportive partner may aid in coping with stress and preventing burnout [8].

As previously reported [45], we did not find differences in the scores of any of the burnout dimensions when comparing nurses and nurse assistants. However, it is likely that such associations are confounded by several factors, including gender, age, workload, salary, and educational level [8]. Thus, because we could only control for gender and age, the (lack of) association reported should be interpreted carefully.

As expected, we found lower DP scores in the recovery room compared with the emergency room/Intensive care unit, as the former cares for fewer patients, who are usually more stable and have a better prognosis.

We found no association between the dimensions of burnout and having an additional job, although this may have resulted from lack of power. Previous studies have yielded mixed results [42], [52].

Thus, our findings are generally consistent with the few previous studies conducted on military nurses, despite the fact that comparisons across studies is complicated by the use of different instruments, reference standards and definitions of burnout, analytic techniques and the different study settings. This coherence greatly supports the validity of our findings.

It is interesting to note that the scores for each of the burnout dimensions reported in our study population were consistently and considerably lower than those reported from American/European populations [47], [55]. The EE and DP scores reported were significantly lower than those reported from the American normative population [55], while the PA scores were significantly higher than the aforementioned standard. Specifically, the cut-off-points for defining the “high” scores of EE, DP and PA (scores in top tertile for EE and DP, and scores in the lower tertile for PA) of the American normative population (EE score ≥27, DP score ≥10 and PA score ≤32) corresponded to the 92.5^th^, 86^th^ and 89^th^ percentile, respectively. This strongly contends that the aforementioned cut-off points for defining a high level of the burnout dimensions may be inappropriate for our study population. Such a discrepancy has also been noted in many previous studies, and supports the recommendation that “reference” cut-off points derived from foreign countries/contexts should not be uncritically adopted [47]. This may be particularly true when studying a population with a different socio-economic, cultural and political context from that prevailing in developed countries.

Given its exploratory nature, this study has some limitations that should be considered when interpreting its results. First, because no reference cut-off scores for the MBI subscales exist for Peruvian nurses, and the concerns about the appropriateness of using foreign reference scores, we analysed the scores for each of the MBI subscales as continuous, precluding the possibility of determining the prevalence of burnout. Nevertheless, this was not an aim of the study, and the analytic approach used avoided the need to rely on “reference” cut-off points of questionable validity in our study population.

Second, we had no information on several occupational covariates potentially associated with burnout, and could not include them in the analysis. However, some of the variables included in the analysis may constitute proxies for such characteristics, and capture part of such information (e.g. department, occupation, having an additional job). Indeed, because the method with which work shifts are programmed in the critical care areas tend to homogenise work demands across the nursing staff, it is unlikely that such factors could account for a considerable variability in the study.

Third, the limited sample size of the study severely restricted the possibility of controlling for additional potential confounders in the multivariable analysis, performing more advanced analyses (such as subgroup comparisons), and may have rendered some of the statistical tests underpowered. However, this was unavoidable given the limited size of the available population (military acute and critical care nursing personnel), even with an excellent response proportion.

Finally, the special setting and characteristics of the nursing population evaluated in the study may complicate the generalizability of the study findings. However, we believe that the study results may reasonably be applied to other military critical care nursing personnel working in large military hospitals in Latin America.

Future confirmatory studies conducted in several military health centres (perhaps from different contexts) may benefit from greater sample sizes (and thus, reach more solid conclusions) and greater generalizability of their findings.

The study findings are important for several reasons: this is one of the first published studies that explored the socio-demographic and occupational correlates of the dimensions of burnout among military in military critical care nurses in Peru, improving our understanding of the condition in this high risk, yet understudied, occupational group. Second, identification of the socio-demographic and occupational factors associated with the dimensions of burnout may aid the identification of high-risk groups, and potentially modifiable risk factors for burnout, a critical step for developing effective preventive strategies for this vulnerable population. Finally, this study underscores important differences which exist in the measurement and experience of burnout in acute and critical care military nurses from a developing country in Latin America, emphasizing the need for further research in this population, in order to characterize the nature of burnout in this context.

In conclusion, this exploratory study suggests that among Peruvian military acute and critical care nursing personnel potential screening and preventive interventions should focus on younger/less experienced nurses/nurse assistants, who are single, have children, or work in the most acute of the critical care departments (e.g. the emergency room or the intensive care unit).
